# Dynamic interfacial tension measurement method using axisymmetric drop shape analysis

**DOI:** 10.1016/j.mex.2018.06.012

**Published:** 2018-06-23

**Authors:** Nikhil Bagalkot, Aly A. Hamouda, Ole Morten Isdahl

**Affiliations:** Department of Energy and Petroleum Engineering, University of Stavanger, Norway

**Keywords:** Dynamic IFT measurement, Dynamic IFT, Pendant drop method, Multiphase, Dynamic density

## Abstract

The current method describes a simple modification to the dynamic and equilibrium interfacial tension (IFT) measurement in a multiphase system (gas-liquid/liquid-liquid) by the Axisymmetric Drop Shape Analysis (ADSA) pendant drop technique. The primary difficulty associated with dynamic IFT measurement by ADSA is providing the appropriate phase densities, especially in a system consisting of gas (CO_2_, methane, and propane) and liquids (water and hydrocarbon). The density of the phases is calculated using a, considering the solubility og gases in liquids, as a function of time. The calculated densities of the phases are then used as inputs in the experiment to measure the IFT at high pressure and temperature PVT-cell.

The method offers benefit such as:

• Straightforward and cost effective as it does not require additional experimental setup (like density meter) or a complicated equation of state.
• The composition of the binary mixtures (mole and mass) and the density changes of the binary mixture due to mass transfer may be obtained as a function of time at fixed pressure and temperature.
• IFT as a function of time is measured by taking into consideration of correct phase density.

## Graphical abstract

**Figure:**
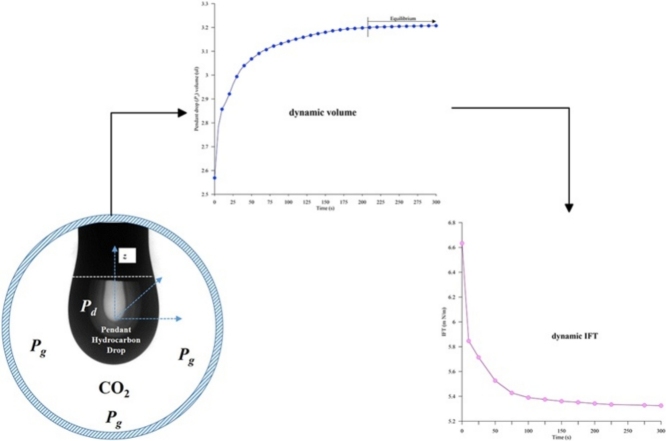

**Specifications Table**

**Table:** 

Subject area	*Select one of the following subject areas:* • *Chemical Engineering* • *Engineering* • *Mathematics*
More specific subject area	*Mass transfer and interfacial science*
Method name	*Dynamic IFT measurement*
Name and reference of original method	Bagalkot, Nikhil, and Aly A. Hamouda. “Experimental and numerical method for estimating diffusion coefficient of the carbon dioxide into light components.” *Industrial & Engineering Chemistry Research* 56.9 (2017): 2359–2374. Zolghadr, Ali, Mehdi Escrochi, and Shahab Ayatollahi. “Temperature and composition effect on CO2 miscibility by interfacial tension measurement.” *Journal of Chemical & Engineering Data* 58.5 (2013): 1168–1175.
Resource availability	Equipment theory: https://www.kruss-scientific.com/services/education-theory/glossary/pendant-drop/ Equipment: https://www.kruss-scientific.com/products/contact-angle/dsa100/drop-shape-analyzer-dsa100/ Software: https://www.kruss-scientific.com/products/advance-software/overview/

## Method background and description

Interfacial tension plays a significant role in numerous engineering applications involving multiphase flow. Measuring the IFT is a crucial part of multiphase systems, there are several methods available like ring method, drop volume method, spinning drop method, bubble pressure method and pendant drop method. In recent years, the pendant drop method has been widely used as an effective method with high accuracy (±0.05 mN/m^2^) [1,2], especially at elevated pressure and temperature. There are several types of equipment available that rely on pendant drop method to estimate the IFT, few of them are IFT-700 (Vinci Technologies), IFT-10-P (Core laboratories), DSA-00 (KRÜSS), and Model-190 (ramé-hart instrument). Most of these use image processing combined with Young-Laplace equation to estimate the IFT.

The primary difficulty associated with IFT measurement by pendant drop mechanism is providing the appropriate densities of the two phases, especially in a system consisting of gas (like CO_2_, methane, and propane) and liquids (water and hydrocarbon). Multiphase systems like CO_2_-hydrocarbon, CO_2_-water/brine, and carbonated water-hydrocarbon are of increasing interest due to their application in petroleum (CO_2_ EOR), environmental (CO_2_ sequestration) and renewable energy (geothermal). When CO_2_ contact liquid (hydrocarbon) it diffuses and dissolves into the liquids, forming a binary mixture. The diffusion of gases into liquids alters the composition of the resulting binary mixture, hence alter the properties like density.

Obtaining the density of the binary mixture is complex, especially at elevated pressures and temperatures and as a function of time. Most of the studies have neglected the density changes due to the solubility effects of dissolved gases in bulk liquids and have used the density of pure fluids instead of the binary mixture [3,4]. While some studies have used separate high pressure and temperature density measuring equipment at equilibrium condition (not dynamic), which complicates the system [5,6], as it requires two different setups. Some studies have even used a complex equation of state model (GERG equation of state (EOS)) [7].

In the present study, a simple and effective method is used to measure the dynamic and equilibrium IFT of the fluid-fluid system with a mass transfer across the interface. Instead of a complicated EOS model or expensive additional instrument, in the present method, the density of changes in the hydrocarbon due to CO_2_ mass trasfer is measured from a combination of experimental and analytical approach, and the obtained density is then used to estimate the IFT by pendant drop technique.

### Principle of IFT measurement

The pendant drop method is an effective and popular means to measure the interfacial tension of liquid-liquid or liquid-gas system. In the pendant drop method, the drop is created from a needle (capillary tube) in a bulk phase (liquid or gas) inside a PVT-cell. The shape of the pendant drop is governed by gravity and the surface/interfacial tension. The IFT is calculated from the shadow of the digital image captured by the camera using the drop shape analysis. The drop shape analysis relies on Young-Laplace equation (Eq. (1)) for calculation of IFT [8,9].

(1) $$\Delta P=\sigma \cdot \left(\frac{1}{r_{1}}+\frac{1}{r_{2}}\right),$$

where ΔP is the pressure across the interface; r_1_ and r_2_ are the principal radii of the pendant drop, and σ is the interfacial/surface tension.

While carrying out an IFT measurement, the scale of the digital image is measured first to get the actual dimension of the pendant drop. Once the scale is obtained, using grey scale analysis, the shape of the drop is then determined. A shape parameter (B) is then adjusted in a numerical method until the calculated drop shape resembles with the actual shape. The interfacial tension may then be calculated from Eq. (2) from the density difference between the *P_g_* and *P_d_* (Δρ=*P_d_* – *P_g_*) and the modified shape parameter (B) [8,10].

(2) $$\sigma =\frac{\Delta \rho gd^{2}}{B},$$

where Δρ is the density difference between the phases; g is the acceleration due to gravity, and d is the maximum horizontal diameter of the unmagnified pendant drop.

From Eqs. (1) and (2) it may be observed that except for density difference (Δρ), the rest of the parameters are calculated by the image processing software. The density of the phases goes as input that the user has to provide. Therefore, even if the software is highly accurate, an inaccurate density input would result in an incorrect IFT. Therefore, the actual density of the phases play a crucial role in estimation of the IFT.

### Materials

In the present study, CO_2_ (PRAXAIR with purity greater than 99%) and n-decane (Merck KGaA with purity 99%) were used as the experimental fluids. *NIST Chemistry Web Book* [11] was the source of density and viscosity measurements for pure substances (n-decane, and CO_2_).

### Experimental setup

**Fig. 1:**
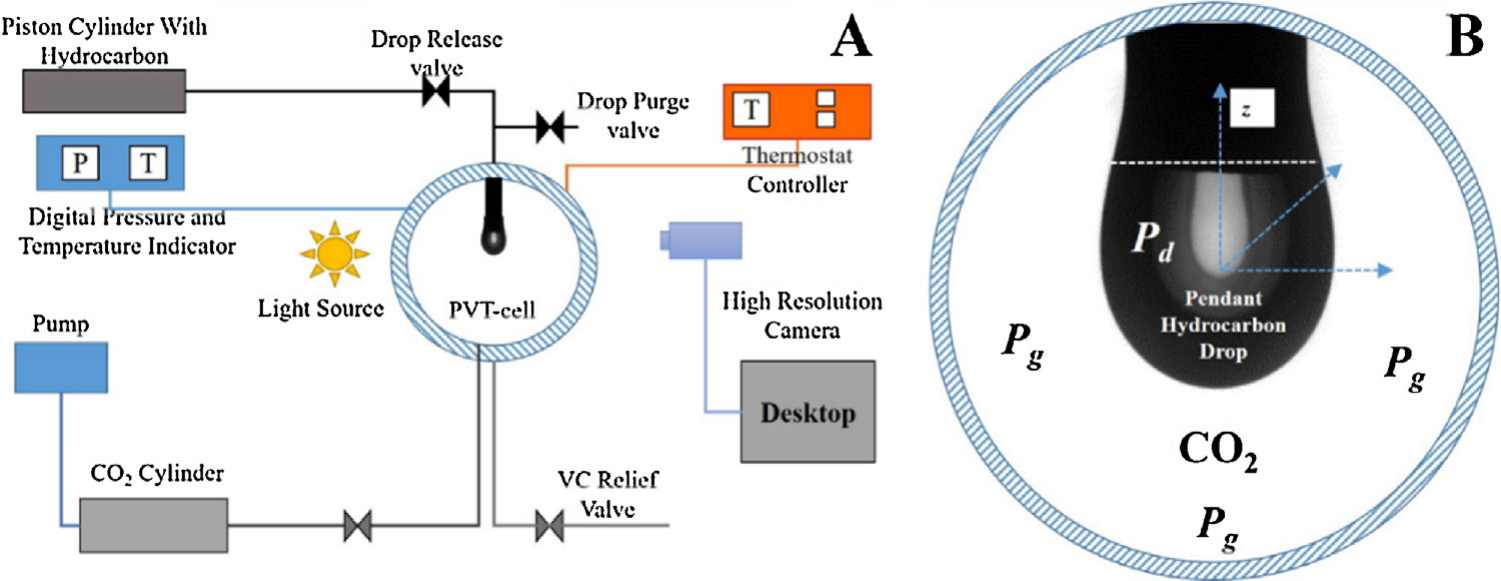
(1A) Schematics of the experimental setup; (1B) Arrangement of the pendant drop in the PVT-cell.

Fig. 1A shows the schematics of the experimental setup. The critical part of the setup is the High-Pressure Pendant Drop Apparatus (PD-E1700 LL-H) (PVT-cell) built by EUROTHECHNICA and KRUSS [12], which has been used to measure the interfacial tension. In Fig. 1A, PVT-cell is corrosion resistant, high-pressure cylindrical chamber (25 ml capacity) having a limiting pressure and temperature of 690 bar and 180 °C respectively. The PVT-cell is a see-through chamber, in which the pendant drop will be created. Fig. 1B shows the arrangement of drop phase (pendant drop, *P_d_*) and the surrounding environmental phase (gas or liquid, *P_g_*) in the PVT-cell. The temperature of the PVT-cell is controlled by a NiCr-Ni thermocouple fitted with a digital indicator. The pressure of the system is maintained externally through a pump (maximum pressure of 32 MPa, GILSON) connected to the gas cylinder. The PVT-cell has a see-through window and is placed between a high-resolution camera (CF03), and a light source. KRUSS DSA 100 (ADVANCE) [13] software is used to analyse the acquired images and compute the *P_d_* volume, and interfacial tension (IFT) at pre-set time steps. Further, details of the experimental setup may be found in Bagalkot and Hamouda [14]. The pressure sensor has an accuracy of ± 0.1 MPa, while the temperature sensor has accuracy of ±0.1 °C at 0 °C to ±0.8 °C at 400 °C, respectively.

## Procedure for measurement of IFT

**Fig. 2:**
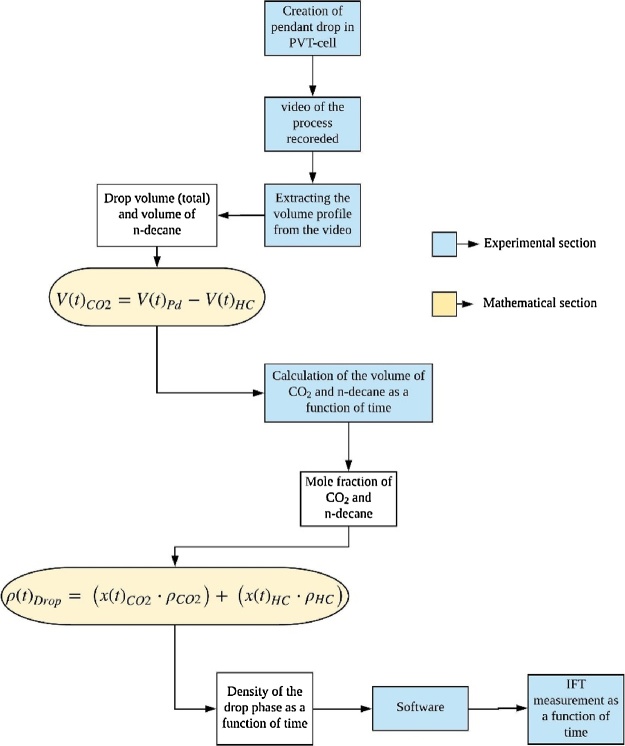
Schematic representation of the process involved in the measurement of IFT.

In the current work the CO_2_-decane system has been taken as the reference system, with CO_2_ being the environmental phase/fluid (*P_g_*) and n-decane the drop phase/fluid (*P_d_*). Fig. 2 shows the schematic representation of the process involved in the measurement of IFT.

**Fig. 3:**
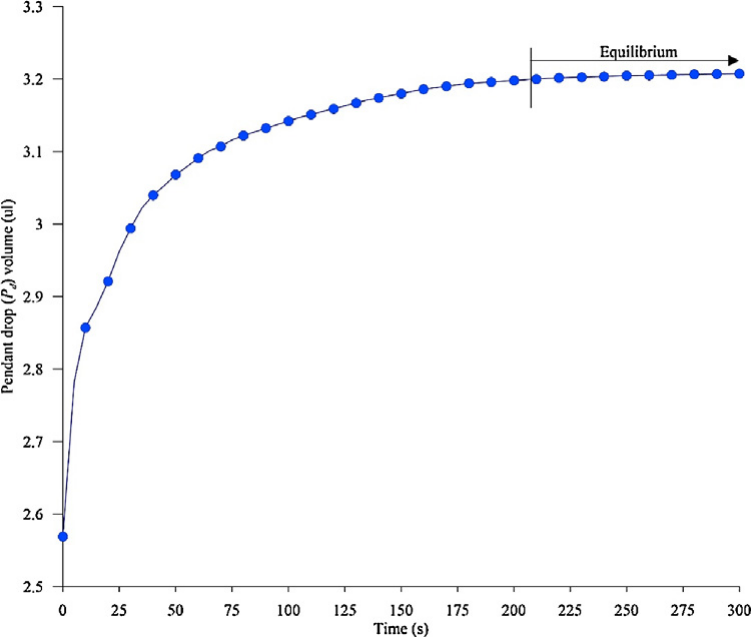
Volume of the pendant drop as a function of time.
5 From the images captured, with the aid of the image analysis software, the volume of the *P_d_* at different time steps will be obtained (Fig. 3).
  At the start of the experiment (time t = 0), the *P_d_* consists solely of n-decane (100% hydrocarbon). With the initiation of the CO_2_ diffusion (t > 0 s), the volume of the *P_d_* increases due to the additional volume of CO_2_. Hence, the volume of the pendant drop (*V_Pd_*) would be a summation of the volume of hydrocarbon (*V_HC_*) and the increase in volume caused by the diffusion of CO_2_ (V_CO2_) in the *P_d_*, as given by Eq. (3).
  (3) $$V_{Pd}(t)=V_{CO2}(t)+V_{HC}$$
  For a fixed temperature and pressure the volume of the n-decane would be same as that during the start of the experiment (since the PVT-cell is closed for any additional decane mass to come in). Hence, in Eq. (3) *V_Pd_* (obtained from the experiment (step 5) and *V_HC_* are known, therefore rearranging Eq. (3) would give the volume of CO_2_ in the pendant drop as given by Eq. (4).
  (4) $$V_{CO2}(t)=V_{Pd}(t)-V_{HC}$$
6 From the acquired volume of CO_2_ (*V_CO2_*) and decane (*V_HC_*) in *P_d_* at every time step (step 5), the mass and moles, and hence, the mole fraction of CO_2_ (*x*_CO2_), and n-decane (*x*_HC_) may be obtained at all time steps.
7 The calculated dynamic mole fraction of CO_2_ (*x*_CO2_) and mole fraction of n-decane (*x*_HC_) was further be used to obtain the density of the *P_d_* consisting of a binary mixture (*ρ_Pd_*) at every experimental time step by Eq. (5) [[15], [16], [17]].
  (5) $$\rho {\left(t\right)}_{Pd}={\left(\left(x{\left(t\right)}_{CO_{2}}\cdot \rho_{CO_{2}})+(x{\left(t\right)}_{HC}\cdot \rho_{HC}\right)\right)}_{P,T},$$
  where *ρ_CO2_* and *ρ_HC_* are the densities of CO_2_ and hydrocarbon in the drop, respectively (obtained from NIST webbook [11]).
8 The density data of the CO_2_ and density of pendant drop consisting of CO_2_+n-decane from Eq. (5) was used as an input to the software to obtain the dynamic and equilibrium IFT of the CO_2_-decane system.

1 The PVT-cell is filled with the environmental fluid at required pressure using the CO_2_ cylinder connected to the pump, which is set at the required pressure as shown in the Fig. 1A. Therefore, at all times the pressure inside the PVT-cell is maintained. Further, the temperature of the PVT-cell is set, which is maintained by a NiCr-Ni thermocouple.
2 Once the PVT-cell consisting of environmental fluid achieves the required pressure and temperature, an n-decane pendant drop (*P_d_*) is created at the end of the capillary tube as shown in the Fig. 1B.
3 As soon as the pendant drop is created, the camera and the ADVANCE software starts to capture the high-resolution digital images of the pendant drop for the analysis.
4 Diffusion of CO_2_ (*P_g_*) into the n-decane (*P_d_*) starts when the fluids come in contact with each other, resulting in a binary mixture of CO_2_+decane. The diffusion of CO_2_ would result in increased volume of *P_d_*. The experiment will continue until the volume of the pendant drop has reached equilibrium (point above which there is no or minimal increment in the volume of the pendant drop) (Fig. 3 at 50 bar 25 °C).

## Validation

As described in Section 1.1, for the pendant drop method the IFT measurement is a function of the density of phases. Therefore, to validate the present method, it would be sufficient to validate the density values calculated from the Eq. (5), with that obtained in literature. Density data of CO_2_+decane binary mixture at 34 bar and 40 °C obtained by Kandil et al. [16] was used to validate the present method. The density of the present work and that form Kandil, et al. [16] were be input into the software for the experiments carried out at 35 bar, and 40 °C with the CO_2_-decane system. The obtained IFT’s were then compared for both of the density inputs (Table 1). It may be observed that both density and obtained IFT of the present method are comparable with Kandil et al. [16], therefore, validating the present method of calculating the density and hence, the IFT.

**Table 1:** Validation of density and IFT of the present model at equilibrium condition.

Study	Density of CO_2_-decane pendant drop (g/ml)	Density of CO_2_ [11] (g/ml)	IFT (m N/m)
Kandil et al. [16]	0.720	0.07087	12.85
Present method	0.711	0.07087	12.53

## Method results and comparison

To demonstrate the importance of correct phase densities (*P_g_* and *P_d_*) in the estimation of IFT, two different methods on density input from the literature were used and compared with the method presented in the current article. All three methods are analysed using the same experiment carried out at 50 bar, 25 °C for a CO_2_-decane system. The details of each of these methods are described below:

*Case 1 (initial density)* [3,4]: This method uses the pure phase density (CO_2_ and decane) and neglecting the density changes due to the diffused gases (CO_2_+decane) in bulk liquids to estimate the IFT.

*Case 2 (equilibrium density)* [2,6]: In this method, the IFT is measured using the equilibrium phase density. The density change due to the solubility of gases was then considered, however, it is done only at equilibrium (final point), and this equilibrium density is used to estimate IFT for the whole process (at all times).

*Case 3 (dynamic density, present method)*: Here the IFT is measured using the corrected density of the phases (*P_g_* (CO_2_) and *P_d_* (CO_2_+decane)) at every time step calculated from Eq. (5), then following the procedure described in section 2.0. The present method improves case-2 as it is capable of calculating the density change of the drop phase (CO_2_+decane) cause by the solubility of the gas, as a function of time. This is unlike in case-2 where only the density of equilibrium is considered. Therefore, the present method reflects the real-time changes in density on IFT, without requiring additional setup as in case-2.

**Fig. 4:**
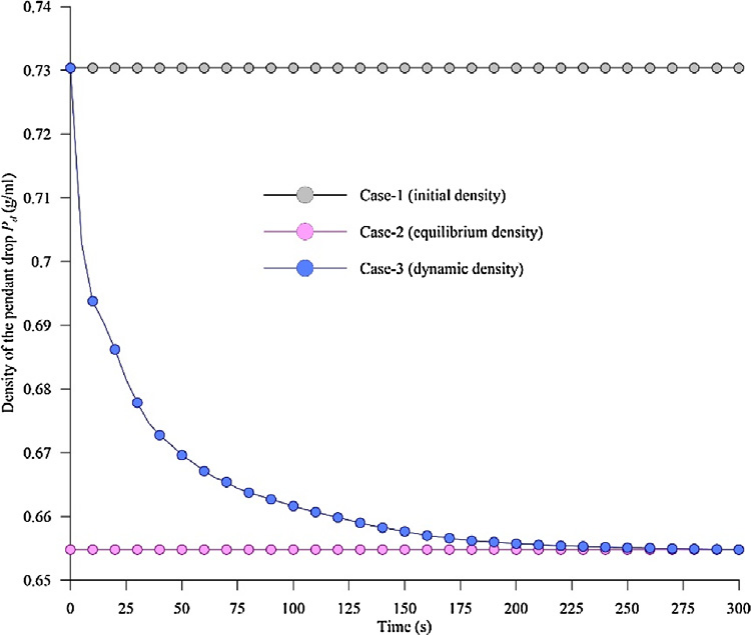
Density of the pendant drop as a function of time for case-1, case-2 and case-3 at 50 bar and 25 °C.

Fig. 4 shows the density of the pendant drop phase obtained from the three cases 1–3 as a function of time at 50 bar and 25 °C for the CO_2_-decane system. The difference in the density vs time profile among the cases (1–3) is evident. The density for case-1 (initial density) and case-2 (equilibrium density) remain constant with time, while the density for case-3 (dynamic density) which represent the actual scenario varies (decreases) with time, depicting changes in density of the drop phase (*P_d_*) due to the solubility of CO_2_ in decane.

**Fig. 5:**
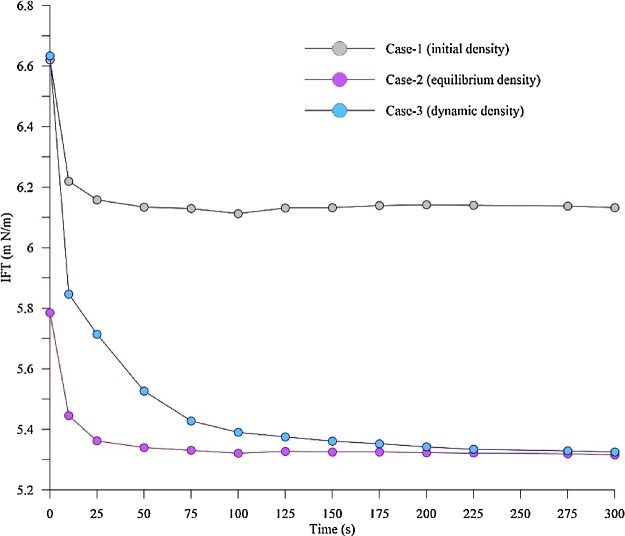
Dynamic IFT CO2-decane system as a function of time for case-1, case-2 and case-3 at 50 bar and 25 °C.

Fig. 5 shows the IFT of the CO_2_-decane system for case 1, case-2, and case-3 at 50 bar and 25 °C. It is clear from the Fig. 5 that different IFT vs time profiles are obtained for the same experiment, emphasising, the dependency of the IFT measurement on the density. Since, both case-1 and case-3 do not consider the dynamic change in the density of pendant drop due to the solubility of a gas in a liquid, the dynamic IFT measured by these methods are different from the one where the solubility effect is considered (case-3). The error in the estimation of IFT varies from a max of 13.4% to min of 0.2% for case-1 and maximum of 14.7% to a minimum of 0.2% for case-2 when compared to case-3. However,the equilibrium IFT for case-2 and case-3 seems to be same or near to eachother. If equilibrium IFT is the focus of the study and not the dynamic natureof the IFT, then applying the case-2 would be fine, as the densities of the phases represent the equilibrium conditions. However, if the dynamic changes in the IFT, as well as the equilibrium IFT, is to be analysed, then case-3 would be the best option (followed in the present study), as the densities of the phases are calculated at different time intervals until equilibrium is reached. Obtaining IFT using the case 1 approach would lead to an error, as the density represent only the initial state of the system, not the equilibrium or the dynamic.

## Drawbacks and recommendations

Although the method presented is simple, reliable, and free from human interference, the major setback would be manually entering the density of phases in the software. The task may seem simple, but to obtain high resolution dynamic data, it requires a lot of entries. Further, if experiments are carried out with sensitivity for both temperature and pressure, the number of entries is multiplied accordingly. An example, a 4-h experiment with dynamic IFT data for every 5 min and sensitivity for two temperatures and three pressures results inn 576 manual inputs (two for each time-step). However, since the process is repetitive, it is straight forward to let computer-scripts perform the task. More importantly, the problem can be eliminated if the software-developers allowed for input of density of phases obtained from the model as either a function of time or as a table with time and density of the phases.
